# Effects of Different Progesterone Doses on the Concentrations of Proinflammatory and Anti-inflammatory Cytokines in Pregnant Women With Threatened Abortion

**DOI:** 10.7759/cureus.19333

**Published:** 2021-11-07

**Authors:** Pınar Kırıcı, Elif Seren Tanrıverdi

**Affiliations:** 1 Obstetrics and Gynaecology, Adıyaman University, Adıyaman, TUR; 2 Microbiology, İnönü University, Malatya, TUR

**Keywords:** randomised clinical trial, tnf-alpha, il-6, il-10, serum progesterone, threatened miscarriage

## Abstract

Background and objective

This study aimed to investigate how different doses of progesterone influence the concentrations of interleukin-6 (IL-6) and tumor necrotizing factor-alpha (TNF-α), which are proinflammatory cytokines, as well as that of IL-10, which is an anti-inflammatory cytokine, in pregnant women with threatened abortion.

Materials and methods

This is a prospective, single-center, randomized controlled trial conducted with 221 patients with a threatened abortion diagnosis. Group 1 consisted of IL-6, IL-10, and TNF-α values in pre-treatment blood samples from 221 patients diagnosed with imminent abortion. Group 2 included 81 patients who received natural oral 100 mg micronized progesterone MP twice a day for two weeks. Group 3 included 83 patients who were administered oral 200 mg of natural micronized progesterone MP twice a day for two weeks. Group 4 included 57 patients who received oral 200 mg of natural micronized progesterone MP twice a day for two weeks, and one depot progesterone was added to the treatment by administering it at a dosage of 500 mg/day intramuscularly.

Results

IL-6 values between groups were lower in group 4 compared to group 3 (p=0.007). When IL-10 values were compared between the groups, the IL-10 ratio was highest in group 4 and lowest in group 2 (p<0.001, p=0.003, p<0.001). When the TNF-α values between the groups were compared, the value in group 4 was decreased compared to groups 1 and 2 (p=0.031, p<0.001). In the logistic regression analysis, the IL-6 value above 12.01 increased the abortion imminens rate 1.01 times, and a TNF-α value above 11.04 increased the abortion imminens rate 1.21 times.

Conclusion

Progesterone used to treat imminent abortion reduces the levels of proinflammatory cytokines, such as IL-6 and TNF-α, while increasing those of anti-inflammatory cytokine IL-10 in proportion to the dose administered. Progesterone can prevent imminent abortion by generating an anti-inflammatory environment.

## Introduction

Threatened abortion or pregnancy loss before the 20th week of pregnancy is the most prevalent type of pregnancy loss, with a prevalence of 15-20% [1-3]. Although chromosomal abnormalities, cervical malformations, systemic disorders, and the presence of pathogenic microorganisms in pregnant women account for 50-70% of early miscarriages, other causes of miscarriage include radiation, chemicals, and drugs [1,4-6]. The corpus luteum is the primary source of progesterone followed by the trophoblast [7]. By inducing changes in the endometrial cavity, progesterone plays a vital role in the onset and continuation of pregnancy [4,8,9]. At the maternal-embryonic interface, progesterone plays a significant role in determining the maternal immune system's response to paternal antigens [10-12]. Many studies have found that low serum progesterone levels are an important test for predicting the risk of imminent abortion [13-15].

This study aimed to investigate how different doses of progesterone influence the concentrations of interleukin-6 (IL-6) and tumor necrotizing factor-alpha (TNF-α), which are proinflammatory cytokines, as well as that of IL-10, which is an anti-inflammatory cytokine, in pregnant women with threatened abortion.

## Materials and methods

Study design

This study was a prospective, single-center, randomized controlled study. It included 221 patients who were treated at the Adiyaman Training and Research Hospital's gynecological outpatient clinic between February 2020 and February 2021 with a pre-diagnosis of threatened abortion. Patients or their legal guardians provided written and signed consent forms to participate in the study.

Ethical approval for this study was obtained from the Adiyaman Training and Research Hospital Ethics Committee for Non-Interventional Clinical Trials (14/01/2020-2020/1-2). The study was conducted in accordance with the Declaration of Helsinki.

Study population

Two gynecologists, each with 10 and 15 years of experience, performed gynecological examinations and ultrasonography of patients presenting with symptoms of imminent abortion. The inclusion criteria were being between the ages of 18 and 40 years, having an intrauterine gestational sac on ultrasonography, carrying an intrauterine fetus with positive fetal heart pulse below 20 weeks or less than 500 g, retroplacental bleeding without cervical dilation, and a history of vaginal spotting/bleeding. The study excluded patients who had used progesterone during the pregnancy, had a known chromosomal defect, had a history of recurrent abortion (three or more consecutive miscarriages), and had pregnancies using assisted reproductive techniques. Accordingly, 221 individuals at risk of miscarriage with pregnancies under 20 weeks or an intrauterine fetus weighing less than 500 g were included in the study.

Group 1 consisted of IL-6, IL-10, and TNF-α values in pre-treatment blood samples from 221 patients diagnosed with threatened abortion. Group 2 included 81 patients who experienced lower-abdominal pain at the time of admission and had spotting on the gynecological examination. These patients received natural 100 mg micronized progesterone MP orally (Progestan®, Koçak Biologicals B.V., Turkey) twice a day for two weeks. Group 3 included 83 patients who presented with lower abdominal pain and had bleeding that did not surpass the use of one pad per day as well as bleeding on gynecological examination. These patients were administered 200 mg of natural micronized progesterone MP orally (Progestan®) twice a day for two weeks. Group 4 included 57 patients with lower abdominal pain, bleeding during vaginal examination, and bleeding that required the use of more than one pad per day. These patients received 200 mg of natural micronized progesterone MP orally (Progestan®) twice a day for two weeks, and one depot progesterone (Proluton® Depot, Bayer Türk Kimya San. Ltd. Şti., Istanbul, Turkey) was added to the treatment by administering it at a dosage of 500 mg/day intramuscularly (Table 1).

**Table 1:** Patient characteristics of groups. n: number; MP: micronized progesterone; im: intramuscular.

Groups	n	Features	Treatment Applications Method
Group 1	221	The values of third groups before treatment	-
Group 2	81	Lower abdominal pain- and spotting	Oral 100 mg MP progesterone/day
Group 3	83	Lower abdominal pain and bleeding <1 pad per a day	Oral 200 mg MP progesterone/day
Group 4	57	Bleeding requires >1 pad a day	Oral 200 mg MP progesterone and 1 500 mg depot progesterone im/day

Measurement Techniques

A total of 5 cc of intravenous blood was obtained from patients diagnosed with threatened abortion before treatment and two weeks after treatment and preserved in an Eppendorf tube at −80 °C. After obtaining informed consent from patients without pathology who visited the outpatient clinic in the first trimester, 5 cc of their intravenous blood was obtained and stored at −80 °C in an Eppendorf tube for the control group.

Blood samples were obtained from the intervention group before and after progesterone treatment. A total of 2 ml of blood samples from each patient were immediately transferred to PreAnalytiX (PAX, Qiagen BD, Valencia, CA) gene blood collection tubes.

Total RNA Isolation

Total RNA was isolated using the RNeasy Mini Kit (Qiagen, Hilden, Germany) from the PAX gene blood collection tubes. From each sample, RNA quantity and purity were determined spectrophotometrically using the MaestroNano Micro-Volume Spectrophotometer (MN-913, Maestrogen, Taiwan) with a measuring range of 2-2000 ng/μL.

Reverse Transcription Complimentary DNA (cDNA) Synthesis

cDNA synthesis was performed using a Quantitect Reverse Transcription Kit (Qiagen, Hilden, Germany). This system consists of two major steps: reverse transcription (RT) and the elimination of genomic DNA. Genomic DNA is eliminated by incubating each RNA sample in a gDNA wipe-out buffer at 42 °C. Following that, RT mix (1 μL Quantiscript Reverse Transcriptase, 4 μL Quantiscript RT Buffer, and 1 μL RT Primer Mix) and target RNA at 1 μg concentration in 20 μL total volume were prepared on ice.

The reaction mix was incubated at 42 °C for 15 minutes and was then kept at 95 °C for three minutes to inactivate RT. The Quantiscript reverse transcriptase used in this system has a high affinity to RNA and was optimized for RNA at concentrations of 10 pg to 1 mg.

Expression of IL-6, IL-10, and TNF-α by RT-PCR

QuantiTect PCR kit with SybrGreen (Qiagen, Hilden, Germany) and RotorGeneQ (Qiagen, Hilden, Germany) was used for real-time polymerase chain reaction (RT-PCR) according to the RT-PCR instrument manufacturer’s recommendations [16] (Table 2).

**Table 2:** Primer sequences used for quantitative PCR with melting-curve analysis in this study.

Gene*	Forward primer sequence (5′–3′)	Reverse (5′–3′) primer sequence (5′–3′)	Ref
GAPDH	CCATGTTCGTCATGGGTGTG	GGTGCTAAGCAGTTGGTGGTG	16
IL-6	TCCACGGCCTTGCTCTTGTTT	GACATCAAGGCGCATGTGAAC	
IL-10	CGCTGTCATCGATTTCTTCCCT	AGGCATTCTTCACCTGCTCCAC	
TNF-α	GCCCATGTTGTAGCAAACCCT	ATGAGGTACAGGCCCTCTGATG	

Statistical analysis

The relative gene expression was determined by means of the 2 −ΔΔCt comparative method with the use of the Gene Globe Data Analysis Center (Qiagen, Hilden, Germany). All data were normalized according to GAPDH housekeeping gene (HKG) mRNA content.

The RNA expression was analyzed in all groups and compared with the results in the fertile control group (calibrator) as follows:

• Control group: ΔCt = Ct (target gene) − Ct (HKG)

• Group 2, group 3, and group 4: ΔCt = Ct (target gene) − Ct (HKG)

• ΔΔCt = ΔCt (group 1) − ΔCt (groups 2, 3, 4)

• Fold ratio = 2 −ΔΔCt

Quantitative data obtained as a result of expression were analyzed groupwise using IBM SPSS Statistics for Windows (Version 22.0. Armonk, NY: IBM Corp). The Shapiro-Wilk test was used to assess data normality. One-way analysis of variance (post hoc test and Bonferroni test) was used in the analysis of the data conforming to the normal distribution, and the Kruskal Wallis test was used in the analysis of non-normally distributed data. A value of p < 0.05 was considered significant.

## Results

Table 2 shows the demographic features of the patients, weeks of pregnancy (6-9 weeks, 9-12 weeks, 12-15 weeks, >15 weeks), amount of bleeding (spotting, wet pad, soaked >1 pad), patient visit intervals, side effects of progesterone, and miscarriage rates groupwise.

Results showed that there was a significant relationship between IL-6, IL-10, and TNF-α values in groups 2, 3, and 4 after progesterone treatment in patients with an imminent abortion diagnosis (Table 3). Post hoc analyses were conducted using these data to identify the values that resulted in a significant difference between the groups.

**Table 3:** Maternal and follow-up characteristics.

	Group 2	Group 3	Group 4
Maternal age (years; mean±SD)	23.7 ± 4.4	27.7 ± 5.3	25.7 ± 3.3
Gestational age (weeks; mean±SD)	7.3 (7.1, 8.9)	7.7 (6.8, 8.9)	7.4 (6.5, 8.3)
The extent of bleeding at presentation (n, %)			
Spotting	77 (%95)	6 (%7.2)	0
Wet pad	4 (%5)	77 (%92.8)	0
Soaked>1 pad	0	0	57 (%100)
The interval between the first and second visit (days; mean±SD)	7.3 ± 1.6	6.3 ± 1.8	4.8 ± 1.8
Progesterone side effects			
Drowsiness	27 (%33.3)	32 (%38.6)	53 (%93)
Giddiness	33 (%40.7)	51 (%6.2)	51 (%89.5)
Nausea	0	42 (%50.6)	49 (%86)
Abdominal bloating	0	33 (%39.8)	54 (%94.8)

When IL-6 values in groups 2, 3, and 4 were compared with those in groups 1, 3, and 4 demonstrated increased IL-6 levels, but groups 1 and 2 did not show significant differences in IL-6 values (p < 0.001, p = 0.043, respectively). When the IL-6 values in group 3 were compared with those in group 4, IL-6 levels in group 4 were found to be lower than those in group 3 (p = 0.007, Table 4).

**Table 4:** Comparison of biochemical measurements by groups*. SD: standard deviation; IL-6: interleukin 6; IL-10: interleukin-10; TNF-α: tumor necrotizing factor-alpha. *Kruskal Wallis test. Group 1: control group; group 2: 100 mg 2 × 1 oral progesterone; group 3: 200 mg 2 × 1 oral progesterone; group 4: one depot progesterone 500 mg/d and oral 100 mg 2 × 1 progesterone in three days.

Group		IL-6	IL-10	TNF-α
Group 1	Mean± SD	14.15±1.46	10.63±2.4	11.55±1.32
Group 2	Mean±SD	12.24±1.91	11.30±1.28	11.43±1.44
Group 3	Mean±SD	10.31±2.26	12.91±2.37	10.41±1.37
Group 4	Mean±SD	9.07±1.63	14.01±1.93	10.01±1.21
p-value		<0.001	<0.001	<0.001

When IL-10 values were compared between the groups, those in groups 3 and 4 were lower compared to those in group 1 (p < 0.001, p = 0.009, respectively), whereas there was no significant difference between groups 1 and 2 (p > 0.05). When IL-10 values in groups 2, 3, and 4 were compared, the IL-10 value was found to be the highest in group 4 and the lowest in group 2 (p < 0.001, p = 0.003, p < 0.001, respectively). The IL-10 value in group 3 was significantly lower than that in group 4 (p = 0.011, Table 4).

TNF-α values ​​were also compared between the groups, and the results showed that TNF-α values ​​in groups 3 and 4 were decreased compared to those in group 1 (p < 0.001, p = 0.031, respectively). There was no statistically significant difference between group 2 and the other groups in terms of TNF-α values (p > 0.05). Moreover, there was no significant difference in TNF-α values between groups 3 and 2 or 4 (p > 0.05). When compared to groups 1 and 2, the TNF-α value in group 4 was lower (p = 0.031, p < 0.001, respectively; Table 5).

**Table 5:** Post hoc one-way ANOVA analysis of biochemical values by groups. SD: standard deviation; IL-6: interleukin 6; IL-10: interleukin-10; TNF-alpha: tumor nekrotizing factor-alpha. *Kruskal Wallis test. Groups with statistically significant differences between them. Group 1: control group; group 2: 100 mg 2 × 1 oral progesterone; group 3: 200 mg 2 × 1 oral progesterone; group 4: 1 depot progesterone 500 mg/d and oral 100 mg 2 × 1 progesterone in 3 days*I: group designated for comparison; J: other groups compared.

Dependent Variable	Group (I)	(J) Group	Average difference (I-J)	p-value
IL-6	Group 1	Group 2	0.10	0.072
		Group 3	0.94*	<0.001
		Group 4	1.27*	0.043
	Group 2	Group 1	0.10	0.072
		Group 3	−0.83^*^	0.037
		Group 4	−1.13^*^	<0.001
	Group 3	Group 1	−0.94*	<0.001
		Group 2	0.83^*^	0.037
		Group 4	0.47^*^	0.007
	Group 4	Group 1	−1.27*	0.043
		Group 2	1.13^*^	<0.001
		Group 3	−0.47*	0.007
IL-10	Group 1	Group 2	−0.44	0.701
		Group 3	−0.76^*^	<0.001
		Group 4	−1.01	0.009
	Group 2	Group 1	0.44	<0.001
		Group 3	0.41^*^	0.003
		Group 4	0.86^*^	<0.001
	Group 3	Group 1	0.76^*^	<0.001
		Group 2	−0.41^*^	0.003
		Group 4	0.61^*^	0.011
	Group 4	Group 1	1.01	0.009
		Group 2	−0.86^*^	<0.001
		Group 3	−0.61^*^	0.011
TNF-alpha	Group 1	Group 2	0.17	0.082
		Group 3	0.82*	<0.001
		Group 4	1.08	0.031
	Group 2	Group 1	−0.17	0.082
		Group 3	0.91	0.075
		Group 4	0.04	0.051
	Group 3	Group 1	−0.82*	<0.001
		Group 2	−0.91	0.075
		Group 4	0.11	0.081
	Group 4	Group 1	−1.08*	0.031
		Group 2	−4.20^*^	<0.001
		Group 3	−0.11	0.081

Table 6 shows the results of a logistic regression analysis used to calculate the imminent abortion risk based on the patients' IL-6, IL-10, and TNF-α levels and progesterone doses. Accordingly, Group 3 had the highest abortion rate, whereas Group 2 had the lowest. While an IL-6 value above 12.01 increased the imminent abortion rate 1.01 times, a TNF-α value above 11.04 increased it 1.21 times. There was no significant difference between other values ​​and imminent abortion risk (Table 6).

**Table 6:** Univariate logistic regression analysis to determine the risk of threatened abortion. OR: odds ratio; CI: confidence interval; IL-6: interleukin 6; IL-10: interleukin-10; TNF-alpha: tumor necrotizing factor-alpha; group 1: control group; group 2: 100 mg 2 × 1 oral progesterone; group 3: 200 mg 2 × 1 oral progesterone; group 4: one depot progesterone 500 mg/d and oral 100 mg 2 × 1 progesterone in three days.

Factors	Univariate analysis		
	OR	95% CI	p-value
IL-6	1.01	1.03–1.14	0.001
IL-10	1.06	0.99–1.12	0.103
TNF-alpha	1.21	0.92–1.27	0.041
Group 2	1.14	1.07–1.95	0.017
Group 3	1.62	1.22–2.15	0.001
Group 4	1.00	0.98–1.01	0.669

## Discussion

In this prospective study, we found that the levels of cytokines in women diagnosed with imminent abortion varied depending on the dose of progesterone they received during their treatment. Proinflammatory cytokines, such as IL-6 and TNF-α, as well as anti-inflammatory cytokines, such as IL-10, have been demonstrated to decrease with an increase in the amount of progesterone administered to women facing the danger of an imminent abortion. Furthermore, elevated IL-6 and TNF-α levels have been linked to an increased risk of threatened abortion.

Although the T helper cell 1(Th)1/Th2 cell ratio peaks in the endometrium during the proliferative phase, it decreases significantly in the secretory phase and reaches its lowest level [17]. During this period, implantation occurs in a pro-inflammatory microenvironment. A low serum progesterone value has been indicated to exclude a healthy pregnancy, and low progesterone has been reported to predict the risk of abortion [14,15]. By stimulating the Th2 response, progesterone contributes to implantation by decreasing the level of inflammatory cytokines and ensuring fetal viability [18].

A previous study showed that Th2 cells were deficient in women with imminent abortion [19]. In a study investigating the levels of Th1 and Th2 cells in women with recurrent abortions and healthy pregnant women, the values of cytokines secreted by Th1 cells were higher in the abortion group, whereas the concentration of cytokines secreted by Th2 cells decreased significantly [20].

The production of proinflammatory cytokines, such as interferon-alpha (IFN), is down-regulated by cytokines such as IL-4, IL-10, and TGF [21]. IFN is at very high levels in patients at risk of premature labor [22]. IFN production has been reported to decrease after progesterone treatment in a previous study [23]. Furthermore, cervical IFN concentrations were shown to decrease in patients at risk of imminent abortion in a recent study [24].

TNF is another pro-inflammatory cytokine associated with Th1 cells. It activates natural killer cells leading to trophoblast apoptosis [25]. Our study demonstrated that the TNF concentration decreased with the administration of progesterone, indicating that progesterone has an inhibitory effect on the proinflammatory microenvironment.

B cells, Th2 cells, macrophages, and monocytes produce the anti-inflammatory cytokine IL-10, which inhibits IFN production. Previous research has shown that IL-10 levels are lower in women who suffer from abortion compared to those in women who are in labor [26]. Various studies have shown that IL-10 release from CD4+ T cells increases and macrophage activity reduces when progesterone and comparable agents are administered [27,28]. Similar to these studies, progesterone administration enhanced IL-10 concentrations in our study.

There are some limitations of our study. First of all, the clinical evaluation of imminent abortion is very difficult and not always reliable. This could lead to bias. Furthermore, because the sample size in this study is small, larger patient groups are needed in future studies.

## Conclusions

As far as we know, this is the first and current literature study comparing the cytokine levels of pregnant women with threatened abortion treated with progesterone at different dosages and in different forms of use. While it decreases the levels of proinflammatory cytokines such as progesterone, IL-6, and TNF-α, which are frequently used in the treatment of threatened abortion, it increases the levels of the anti-inflammatory cytokine IL-10 in proportion to the administered dose. It has been shown that its effect on anti-inflammatory-pro-inflammatory cytokine levels contributes to its therapeutic role, and it has also been shown that progesterone use and the dose may affect these values. So progesterone can prevent possible abortion by creating an anti-inflammatory environment.
